# *Borrelia turcica *in *Hyalomma aegyptium *ticks in Romania

**DOI:** 10.1186/1756-3305-7-S1-P6

**Published:** 2014-04-01

**Authors:** Z Kalmár, G D'Amico, IA Matei, AI Paștiu, DI Mărcuţan, MO Dumitrache, AD Mihalca

**Affiliations:** 1 Department of Parasitology and Parasitic Diseases, University of Agricultural Sciences and Veterinary Medicine Cluj-Napoca, Cluj-Napoca, Romania

## 

*Testudo graeca *tortoises are distributed in the south-eastern part (Dobrogea region) of Romania. *T. graeca *is a potential host for the three-host ticks, *Hyalomma aegyptium*. *H. aegyptium *ticks are important from epidemiological point of view as they constitute potential reservoirs for numerous zoonotic bacterial pathogens (*Anaplasma phagocytophilum*, *Ehrlichia ca*nis, *Coxiella burnetii*). However, *H. aegyptium *was reported to host less studied bacteria, non-Lyme members of genus *Borrelia*. Despite its relatively wide distribution range, the extent of co-distribution of ticks with these bacteria was not investigated in detail. The aim of the present study was to evaluate *H. aegyptium *engorged ticks collected from tortoises in south-eastern Romania for the presence of non-Lyme *Borrelia*. Between 2008 and 2013, 448 *H. aegyptium *ticks were collected from 45 *T graeca *tortoises located in Dobrogea region in Romania. DNA extraction was performed individually from each tick using a commercial kit. For the total 78 (17.4%) *Borrelia *spp. positive ticks, PCR analysis targeting the intergenic spacer 5S-23S region, *glpQ*, respectively *gyrB *genes, and further sequencing was performed for the further identification. Sequences of *gyrB *and *glpQ *genes showed 99%-100% similarities with reptile-associated *Borrelia turcica*. The most frequently infected stages were males (10.7% of the total males examined or 61.5% from the total infected ticks) followed by females (5.36% of the total females examined or 31% from the total infected ticks) and nymphs (1.34% of the total nymphs examined or 7.7% from the total infected ticks). This is the first report of *Borrelia turcica *in Romania.

This research was supported by grant CNCSIS IDEI PCCE 7/2010 and IDEI PCE 236/2011.

